# A Multidimensional Construct of Religiosity Among Baby Boomers and Trajectories of Social Attitudes

**DOI:** 10.1093/geroni/igab046.1951

**Published:** 2021-12-17

**Authors:** Joonsik Yoon, Woosang Hwang, Maria Brown, Merril Silverstein

**Affiliations:** 1 Syracuse University, Syracuse, New York, United States; 2 Syracuse University, Syracuse, New York, New York, United States

## Abstract

Although a number of studies have examined relationships between religiosity and social attitudes, less is known about how these relationships change over the life course using a multidimensional construct of religiosity among Baby Boomers. A multidimensional construct of religion allowed us to take a more person-centered approach to religiosity, whereby we examine the association between Baby Boomers with different types of religiosity and the trajectories of their political and gender role attitudes over a period of transition from early to later adulthood. We selected 798 young-adult Baby Boomers from the 1971 wave (mean age: 19 years) of the Longitudinal Study of Generations (LOSG) and tracked their political and gender role attitudes through until the 2016 wave (mean age: 64 years). Using latent class analysis, we identified four latent religious typologies: strongly religious, weakly religious, liberally religious, and privately religious. We found that Baby Boomers in the strongly religious class reported the most conservative political and gender role attitudes among the four classes over this period of transition. Baby Boomers in the privately religious class were conservative in their political and gender role attitudes than those in the weakly religious class. The liberally religious group generally reported the second most conservative political attitudes among the four identified groups, but reported the least conservative gender role attitudes of the four groups. Findings suggest that early religiosity may serve as a significant predictor affecting political and gender role attitudes throughout the adult life course.

